# Postmortem Analysis 35 Months after Magnetic Resonance-Guided Focused Ultrasound Thalamotomy for Essential Tremor

**DOI:** 10.5334/tohm.1013

**Published:** 2025-08-28

**Authors:** Saachi Jhandi, Lubdha Shah, Henrik Odéen, Lorraina Robinson, Viola Rieke, Qinwen Mao, Heather Wisner, Josue Avecillas-Chasin, Shervin Rahimpour

**Affiliations:** 1 Spencer Fox Eccles School of Medicine, University of Utah School of Medicine, Salt Lake City, Utah, USA; 2 Department of Radiology and Imaging Sciences, University of Utah, Salt Lake City, Utah, USA; 3 Department of Pathology, University of Utah, Salt Lake City, Utah, USA; 4 Department of Neurosurgery, University of Nebraska, Lincoln, Nebraska, USA; 5 Department of Neurosurgery, Clinical Neurosciences Center, University of Utah, Salt Lake City, Utah, USA

**Keywords:** Essential tremor, MRgFUS thalamotomy, focused ultrasound, demyelination, case report

## Abstract

**Background:**

Magnetic resonance-guided focused ultrasound (MRgFUS) thalamotomy is an emerging, non-invasive treatment for essential tremor (ET). However, postmortem data on the long-term neuropathological effects are limited.

**Case report:**

An 86-year-old man with refractory ET underwent MRgFUS thalamotomy. Tremor improved by 95% and remained controlled until his death 35 months later. Postmortem MRI and neuropathologic analysis showed localized disruption of the dentatorubrothalamic tract and demyelination near the treatment site with preserved neuronal integrity.

**Discussion:**

This is the first postmortem analysis of MRgFUS thalamotomy 35 months after procedure. Findings confirm sustained tremor relief associated with selective demyelination. The lesion remained well-defined without expansion, supporting MRgFUS as a precise and safe treatment for ET.

**Highlights:**

This paper presents the first long-term (35-month) postmortem analysis of MRgFUS thalamotomy demonstrating sustained clinical efficacy. Postmortem MRI confirmed that the lesion remained localized to the original thalamotomy site, with focal disruption of the dentatorubrothalamic tract. Neuropathological examination revealed selective demyelination in the posterior thalamus near the treatment site, without evidence of neuronal loss. These findings support the long-term safety, precision, and durability of MRgFUS as a non-invasive therapeutic option for essential tremor.

## Introduction

Magnetic resonance-guided focused ultrasound (MRgFUS) thalamotomy has emerged as an incisionless therapy for refractory essential tremor (ET) [1,2]. However, neuropathological studies on the long-term effects of MRgFUS on lesioned brain tissue are limited. Only two previous studies have investigated MRgFUS in postmortem settings, both of which focused on patients with Parkinson disease with a relatively short time to death (10 days and 7 months) [3,4]. Additional postmortem analyses after MRgFUS are needed to understand the neuropathological effects of this procedure in relation to temperature rise during treatment, magnetic resonance imaging (MRI) findings, and long-term clinical efficacy. We performed postmortem analysis of a patient with ET who underwent MRgFUS thalamotomy with a follow-up period of 35 months before death.

## Case Report

The patient was an 86-year-old man with a 23-year history of bilateral hand tremor diagnosed as ET. He had persistent functional impairment despite medical therapy with propranolol and primidone trialed for multiple years. His minimum tremor disability score was 2 on the Clinical Rating Scale for Tremor, indicating at least moderate functional impairment affecting his ability to perform fine motor tasks and self-care independently. On patient survey he stated that his tremor significantly interfered with activities of daily living, including making handwriting, using utensils, and buttoning clothing difficult.

Because the patient was not a candidate for deep brain stimulation (due to advanced age) and had a personal preference against surgery, he elected to undergo MRgFUS thalamotomy. Preoperative MRI with T2 weighting and diffusion weighting confirmed no contraindications, and the target for lesioning was the left ventral intermediate nucleus of the thalamus.

The procedure was performed in February 2021 using the Exablate Neuro system (Insightec Ltd., Tirat Carmel, Israel). A stereotactic frame was placed, and sonications were guided by real-time MR imaging. A total of 11 sonications were delivered at energy levels ranging between 1350 and 4946 J. Intraoperative temperature elevations between 49°C and 61°C were recorded. The first four sonications were used for alignment of the FUS. Upon alignment, five additional sonications were performed. Between sonications, tremor reduction and potential side effects such as facial numbness were assessed, and adjustments were made accordingly. The final two sonications resulted in sustained tremor reduction without immediate side effects.

**Figure 1:**
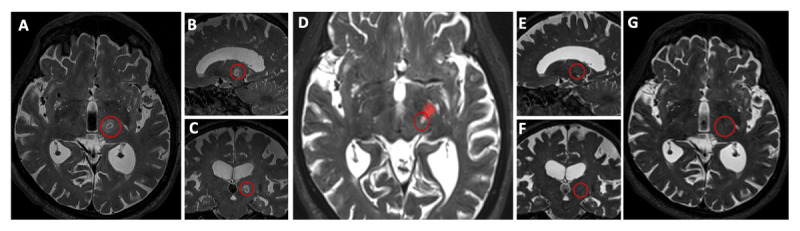
**(A–C)** Axial, sagittal, and coronal high-resolution T2-weighted MRI acquired 3 days after magnetic resonance-guided focused ultrasound thalamotomy demonstrate a hyperintense lesion with central hypointensity, consistent with necrosis and surrounding edema. **(D)** Diffusion tractography overlay reveals proximity of the lesion to the corticospinal tract. **(E–G)** Follow-up imaging at 3 months shows decreased lesion size on axial, sagittal, and coronal views, respectively.

On day one after the procedure, high-resolution three-dimensional T2-weighted imaging showed a 7-mm hyperintense lesion in the thalamus with a hypointense core suggestive of necrosis surrounded by mild edema (Figure 1A–C). Diffusion-weighted imaging at the same time point showed hyperintensity with apparent diffusion coefficient isointensity correlating with reduced diffusivity. Diffusion tractography revealed the lesion’s proximity to the corticospinal tract (Figure 1D). At 3-month follow-up, MRI demonstrated significantly reduced lesion size consistent with expected tissue response (Figure 1E–G).

Postoperatively, the patient experienced transient gait disturbances that resolved within three months. He reported a 95% reduction in tremor severity, with significant improvement in activities of daily living including ability to write, use utensils independently, and dress. Clinical follow-up at six months, one year, and two years confirmed continued tremor suppression without side effects. The patient died from unrelated causes 35 months after treatment. The patient’s family consented to the postmortem examination and publication of his case and images. As a single-patient case study, institutional review board approval was not required.

**Figure 2:**
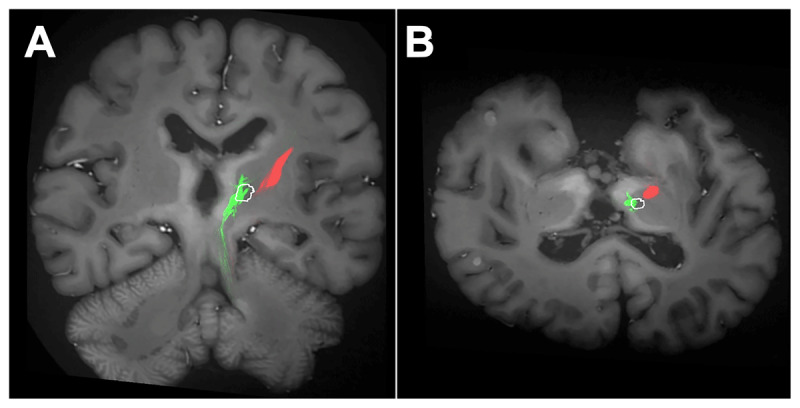
**(A)** Coronal postmortem tractography reveals preserved corticospinal tract (red) and focal disruption of the DRTT (green) at the lesion site (lesion location outlined with white line). **(B)** Axial postmortem tractography confirms the spatial overlap of the DRTT with the lesion and shows the preserved surrounding fibers and corticospinal tract (lesion location outlined with white line).

Postmortem MRI, including diffusion tensor imaging, was performed on the ex vivo brain to evaluate the MRgFUS-induced lesion. The lesion remained localized to the initial site of the thalamotomy, with no evidence of lesion expansion (Figure 2). Tractography demonstrated focal disruption of the dentatorubrothalamic tract (DRTT) fibers at the lesion site correlated with the patient’s clinical improvement.

**Figure 3:**
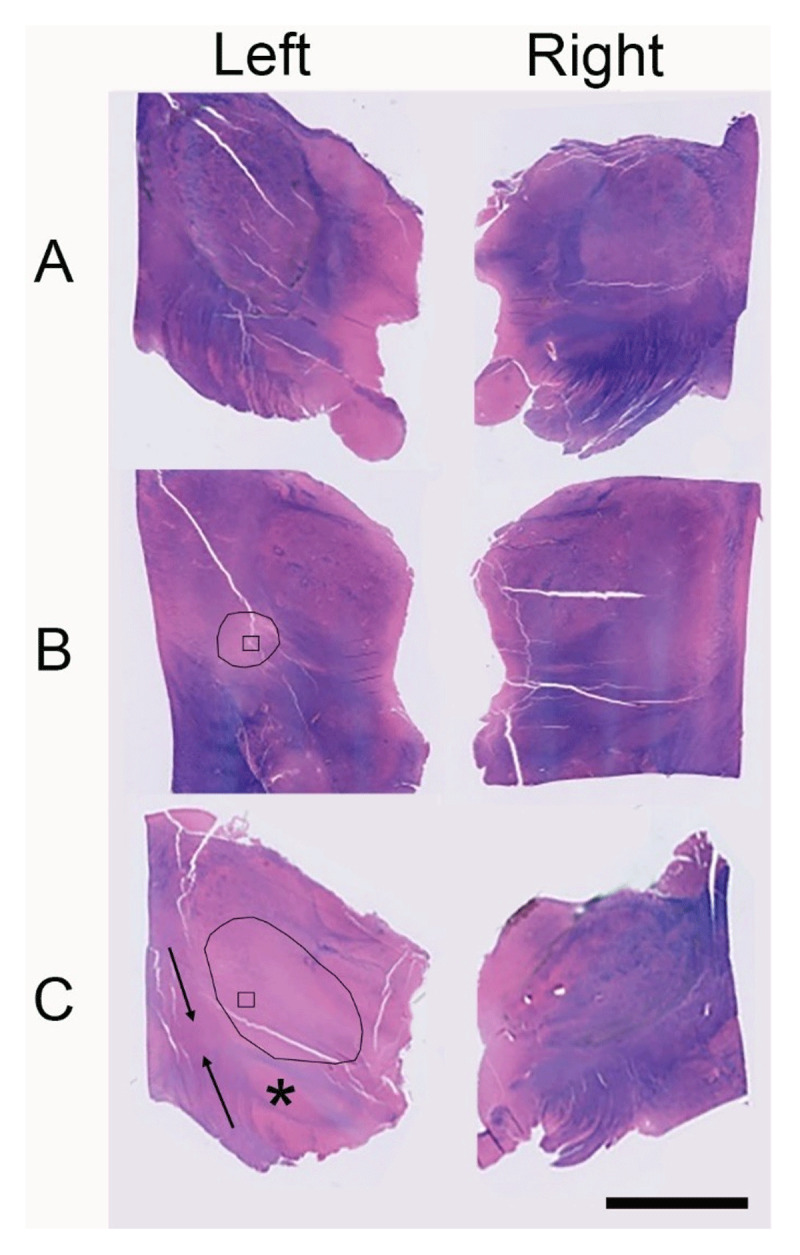
**Luxol fast blue (LFB)-stained sections of the thalamus from both the left and right hemispheres**. Sections progressing from anterior to posterior **(A–C)** were fully submitted for histopathological evaluation. On the left side in **B** and **C**, an area of demyelination corresponding with the magnetic resonance-guided focused ultrasound–treated region is revealed, outlined with black lines. Within this region, black squares indicate the specific area from which the left thalamus images in Figure 4 were obtained. The subthalamic nucleus (*) also appears pale on LFB staining. Adjacent regions of the corticospinal tract (arrows) exhibit demyelinating changes as well. Scale bar: 10 mm.

**Figure 4:**
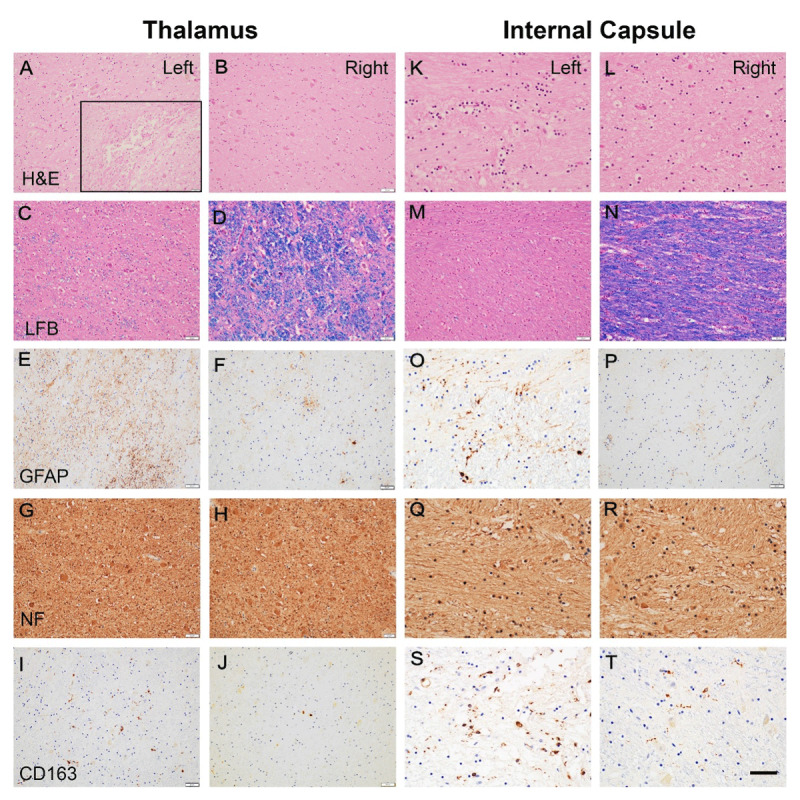
**Histopathological evaluation of the posterior thalamus (A-J) and adjacent internal capsule (K-T)**. The posterior thalamus shows mild vacuolation on the left side without significant neuronal loss on hematoxylin and eosin staining **(A)**, corresponding with the boxed area of Figure 3C (left); the right side at the same level appears normal **(B)**. The inset in A, taken from the boxed area of Figure 3B (left), reveals a small cavitary lesion measuring approximately 1 mm. Luxol fast blue staining shows prominent demyelination on the left **(C)** and preserved myelin on the right **(D)**. Glial fibrillary acidic protein staining demonstrates moderate gliosis on the left **(E)** compared with mild gliosis on the right **(F)**. Neurofilimant staining indicates intact axons bilaterally **(G, H)**. CD163 staining reveals a slightly increased number of macrophages on the left **(I, J)**. The adjacent internal capsule **(K–T)** also exhibits more pronounced demyelination **(M, N)**, increased gliosis **(O, P)**, and greater macrophage presence **(S, T)** on the left compared with the right. Scale bar: 50 µm for all images.

Neuropathological examination of the brain revealed no evidence of generalized edema, infarction, or hemorrhage in the cerebral hemispheres, thalamus, cerebellum, or brainstem. Serial coronal sections of the thalamus appeared grossly unremarkable. Both thalami were entirely submitted for histopathological evaluation. Luxol fast blue staining of the thalamus demonstrated normal myelination in anterior section (Figure 3A) with prominent demyelination appearing in the left posterior thalamus (Figure 3B, C), extending into the adjacent subthalamic region and internal capsule (Figure 3C). Based on these findings, detailed histologic assessment focused on the posterior thalamus (Figure 4). Hematoxylin and eosin staining shown in Figure 4A revealed a 1-mm cavitary lesion indicative of remote necrosis in the area corresponding with the boxed area in Figure 3B (left) and the FUS lesion identified in Figure 2. The surrounding tissue exhibited mild vacuolation without overt neuronal loss and marked demyelination confirmed by luxol fast blue staining, whereas the corresponding region on the right side appeared normal. Glial fibrillary acidic protein immunostaining demonstrated moderate gliosis on the left, in contrast to minimal gliosis on the right. Neurofilament (NF) staining showed preserved axonal integrity bilaterally. CD163 immunostaining revealed a mild increase in macrophage infiltration on the treated (left) side. In the adjacent internal capsule, more extensive demyelination, along with mildly increased gliosis and macrophage infiltration, was observed on the left compared with the right, consistent with secondary involvement of nearby white matter tracts (Figure 4). The adjacent subthalamic nucleus exhibited changes similar to those seen in the thalamus, including marked demyelination without neuronal loss, moderate gliosis, mildly increased macrophage infiltration, and preserved axonal architecture.

## Discussion

MRgFUS thalamotomy has emerged as a non-invasive alternative for patients with medication-refractory essential tremor, demonstrating significant symptom reduction and improved quality of life [1,2]. Although prior studies have characterized lesion evolution using imaging, the histopathological consequences of MRgFUS beyond the first year remain largely unknown [3,4]. This is the longest follow-up of a patient treated with MRgFUS with postmortem data available, offering direct neuropathological validation of lesion integrity, structural specificity, and long-term safety.

At 35 months after treatment, the lesion remained well-demarcated within the ventral intermediate nucleus with no evidence of expansion on postmortem MRI. These findings corroborate prior imaging studies suggesting that MRgFUS lesions stabilize over time without progressive pathology [5,6,7]. Early MRI findings after treatment demonstrate temporary necrosis and surrounding edema at the lesion site [5,6]. Our postmortem analysis at 35 months confirms the resolution of these acute changes, with the lesion maturing into a well-demarcated area without ongoing tissue damage.

Neuropathological examination identified a 1-mm remote necrotic lesion in the left posterior thalamus, consistent with healing and resolution of the MRgFUS lesion. Interestingly, despite the absence of overt neuronal loss, demyelination was observed in adjacent structures of the treatment site, including the surrounding posterior thalamic tissue, internal capsule, and subthalamic nucleus. These findings support the hypothesis that MRgFUS primarily affects white matter pathways involved in tremor generation [3,4,8]. However, the potential for progressive demyelination and its clinical significance warrant further investigation.

Nevertheless, the absence of significant gliosis or neuronal loss reinforces MRgFUS as a targeted intervention with a favorable safety profile. This contrasts with radiofrequency ablation and stereotactic radiosurgery, which have been found to induce tissue necrosis and collateral damage in cardiac tissue and in porcine brain models, respectively [8,9,10]. MRgFUS appears to achieve therapeutic efficacy through precise disruption of tremor circuitry while sparing adjacent structures. The stability of the lesion observed at 35 months further supports the durability of the treatment outcomes and the lack of late-stage complications, aside from localized demyelination of uncertain clinical significance.

Clinically, the patient’s sustained 95% tremor reduction over nearly three years aligns with long-term functional outcomes reported in MRgFUS trials [1,2,5]. Although transient gait disturbances were noted postoperatively, these resolved within three months, consistent with prior studies showing that most adverse effects are mild and self-limited [5,7]. The absence of delayed neurological deterioration in postmortem analysis further underscores that MRgFUS is a safe, long-term intervention. However, it is important to note that the patient had a mild tremor and our data are limited by infrequent follow-up.

This study provides critical neuropathological evidence that MRgFUS is a precise and effective intervention for treatment of medically refractory ET. Further postmortem studies are needed to confirm whether similar histopathologic patterns are observed across larger cohorts and whether specific lesion characteristics correlate with treatment response.

## References

[1] Elias WJ, Lipsman N, Ondo WG, et al. A Randomized Trial of Focused Ultrasound Thalamotomy for Essential Tremor. N Engl J Med. 2016;375(8):730–739. DOI: 10.1056/NEJMoa1600159 27557301

[2] Elias WJ, Huss D, Voss T, et al. A Pilot Study of Focused Ultrasound Thalamotomy for Essential Tremor. N Engl J Med. 2013;369(7):640–648. DOI: 10.1056/NEJMoa1300962 23944301

[3] Blitz SE, Torre M, Chua MMJ, Christie SL, McDannold NJ, Cosgrove GR. Focused Ultrasound Thalamotomy: Correlation of Postoperative Imaging with Neuropathological Findings. Stereotact Funct Neurosurg. 2023;101(1):60–67. DOI: 10.1159/000527269 36696893 PMC9981195

[4] Koga S, Ishaque M, Jeffrey Elias W, Shah BB, Murakami A, Dickson DW. Neuropathology of Parkinson’s disease after focused ultrasound thalamotomy. Npj Park Dis. 2022;8(1):59. DOI: 10.1038/s41531-022-00319-6 PMC9098516 35550514

[5] Zur G, Lesman-Segev OH, Schlesinger I, et al. Tremor Relief and Structural Integrity after MRI-guided Focused US Thalamotomy in Tremor Disorders. Radiology. 2020;294(3):676–685. DOI: 10.1148/radiol.2019191624 31909701

[6] Blitz SE, Chua MMJ, Ng P, et al. Longitudinal MR imaging after unilateral MR-guided focused ultrasound thalamotomy: clinical and radiological correlation. Front Neurol. 2023;14:1272425. DOI: 10.3389/fneur.2023.1272425 37869137 PMC10587555

[7] Kapadia AN, Elias GJB, Boutet A, et al. Multimodal MRI for MRgFUS in essential tremor: post-treatment radiological markers of clinical outcome. J Neurol Neurosurg Psychiatry. 2020;91(9):921–927. DOI: 10.1136/jnnp-2020-322745 32651242

[8] Elias WJ, Khaled M, Hilliard JD, et al. A magnetic resonance imaging, histological, and dose modeling comparison of focused ultrasound, radiofrequency, and Gamma Knife radiosurgery lesions in swine thalamus. J Neurosurg. 2013;119(2):307–317. DOI: 10.3171/2013.5.JNS122327 23746105

[9] Zaer H, Glud AN, Schneider BM, et al. Radionecrosis and cellular changes in small volume stereotactic brain radiosurgery in a porcine model. Sci Rep. 2020;10(1):16223. DOI: 10.1038/s41598-020-72876-w 33004849 PMC7529917

[10] Deneke T, Khargi K, Müller KM, et al. Histopathology of intraoperatively induced linear radiofrequency ablation lesions in patients with chronic atrial fibrillation. Eur Heart J. 2005;26(17):1797–1803. DOI: 10.1093/eurheartj/ehi255 15855195

[11] Connectome – HCP Protocols. Accessed November 1, 2024. https://www.humanconnectome.org/hcp-protocols

